# Strategic Priorities for Research on Antibiotic Alternatives in Animal Agriculture—Results From an Expert Workshop

**DOI:** 10.3389/fvets.2019.00429

**Published:** 2019-11-29

**Authors:** Timothy Kurt, Nora Wong, Heather Fowler, Cyril Gay, Hyun Lillehoj, Paul Plummer, H. Morgan Scott, Karin Hoelzer

**Affiliations:** ^1^Foundation for Food and Agriculture Research, Washington, DC, United States; ^2^Pew Charitable Trusts, Washington, DC, United States; ^3^National Pork Board, Clive, IA, United States; ^4^Agricultural Research Service, U.S. Department of Agriculture, Beltsville, MD, United States; ^5^College of Veterinary Medicine, Iowa State University, Ames, IA, United States; ^6^Department of Veterinary Pathobiology, Texas A&M University, College Station, TX, United States

**Keywords:** antibiotic resistance, animal agriculture, antibiotic alternatives, research prioritization, evaluation framework

## Abstract

The emergence, spread, and expansion of antibiotic resistance and increasing restrictions on the use of antibiotics in animal agriculture have created a need for efficacious alternatives that remains unmet. Prioritizing research needs in the development of alternatives is key to ensuring that scarce research resources are dedicated to the most promising approaches. However, frameworks to enable a consistent, systematic, and transparent evaluation of antibiotic alternative candidates are lacking. Here, we present such an evaluation framework.

## Introduction

Traditional antimicrobial drugs, or antibiotics^1^, are critical tools to promote human and animal health, yet their efficacy is increasingly threatened by antibiotic resistance^2^. Any exposure to antibiotics can select for resistant bacteria; therefore, their use in all settings must be carefully managed (1). In response to this global public health challenge and growing consumer concerns about food production practices, increasing numbers of food companies are voluntarily limiting the use of antibiotics in their supply chains (2).

For the purpose of this study, and consistent with other established definitions [see for instance (3, 4)], alternatives to antibiotics were broadly defined as any substance that can prevent the need for or be substituted for antimicrobial drugs. This includes a wide variety of substances including microbial-derived products (e.g., probiotics, bacteriophages, and bacteriophage-derived products), phytochemicals (e.g., essential oils), immune-derived products (e.g., antimicrobial peptides, immunomodulators), vaccines, enzymes, metals, minerals, and innovative animal drugs. While many of the currently available alternatives enhance animal health and thus reduce the need for antibiotics, they cannot fully replace them. The need for effective alternatives that can more predictably prevent, control or treat disease has remained largely unmet (5)^3^.

Public and private sector funding for research on antibiotic alternatives in animal agriculture is scarce (6). Prioritization is needed to ensure limited resources are dedicated to the most promising and impactful research areas and potential candidates (6). Ideally, the success or failure of an antibiotic alternative would be predictable early during the research and development (R&D) process. However, products may fail at many stages, including after they are fully commercialized. A framework to evaluate antibiotic alternatives early in R&D and enable the consistent and transparent prioritization of investments is sorely needed. This manuscript summarizes the outcomes of an expert workshop organized to address this need.

## Workshop Goals and Objectives

A pre-workshop survey of over 40 experts in animal agriculture identified antibiotic alternatives as the top research priority related to antibiotic stewardship, prompting its selection as the workshop topic (details available upon request). For the purposes of the workshop, antibiotic alternatives were defined broadly as animal feed additives (e.g., phytochemicals, pre- and pro-biotics, organic acids) as well as animal drugs (e.g., immune modulators) and veterinary biologics (e.g., novel vaccines, antibodies) that prevent, control, or treat infectious diseases. In contrast, management practices such as nutritional or backgrounding strategies, improvements in housing, or more stringent biosecurity were excluded from the discussion.

For the workshop, a panel of 23 subject matter experts from academia, industry, governmental, and non-governmental organizations convened for a 1 day in-person meeting in December 2018. The goal was to identify strategic priorities for funding research and development on antibiotic alternatives in animal agriculture. The workshop explored factors critical to the success or failure of new antibiotic alternatives and identified associated data gaps and research needs that, if addressed, could help grow the pipeline of safe and effective product candidates. The workshop consisted of two facilitated discussions to reach consensus on key factors important in the evaluation of research approaches and funding decision-making for antibiotic alternatives. This manuscript highlights key themes that emerged during the workshop and, in certain instances, develops them further. All workshop participants were given an opportunity to review this manuscript before publication.

## Predicting Success or Failure of an Antibiotic Alternative

Successful antibiotic alternatives solve a substantial real-world infectious disease problem and provide an economic and animal health benefit. An entity or entities must be willing to invest in scientific research to bring the product to market, and someone must be willing to purchase and use it. However, that alone is not sufficient for successful adoption. The ability of farmers and veterinarians to use the product relies on additional factors such as the logistics of delivery and storage and whether the product aligns with their own and their customers' values and expectations. Ultimately, many factors influence whether an alternative is successful. Table 1 provides a framework for evaluating the potential success of an antibiotic alternative candidate, starting with an assessment of overall economic viability, followed by a more in-depth assessment of specific risks to product success.

**Table 1:** Framework for evaluating the success of an antibiotic alternative.

**Framework**	**Check-list items**
1.Overall economic viability	
a. Expected project costs	- Product development • Research and Development (R&D) costs •Probability of regulatory approval success •Other feasibility considerations (e.g., Intellectual Property, manufacturability, existing data & models) - Product manufacturing and sales •Cost of materials •Sales and distribution, etc.
b.Expected product revenue	
i Market predictions	- Market size •Number of farms affected & geographic distribution •Disease incidence/prevalence on affected farms •Probability of treating affected animals •Short term economic/animal health impacts •Long-term impacts on animal productivity •Other related impacts (e.g., trade restrictions) -Market characteristics • Market accessibility •Industry structure •Global regulatory landscape •Existing market segmentation • Predicted market growth
ii Product-specific predictions	-Expected return on investment (ROI) for livestock producer/veterinarian •Animal health & productivity improvements • Number needed to treat (NNT) to impact one animal vs. number needed to harm (one animal, or person in case of public health) •Other benefits (e.g., enhanced market access) -Product competitiveness compared to substitutes
2.Specific project risks	
a. Product safety	- Food safety - Target animal safety - Microbial safety - Environmental safety
b. Product efficacy	- Effect type and size - Consistency under real-world conditions - Fitness for purpose
c. Product acceptability	
iii Farmers and veterinarians	- Product perception/mechanism of action - Attitudes, beliefs, perceived behavioral constraints - Trust in the product's consistent efficacy - Product performance relative to expectations
iv Society/consumers	- Consumer acceptance - Ease of explanation
d. Product practicality/ease of use	- Compatibility with current production practices - Administration mode (route, frequency, etc.) - Associated costs (e.g., labor costs, withdrawal times)

### Assessing the Economic Viability of the Project

Profitability is foundational to the success of an antibiotic alternative; farmers and veterinarians cannot adopt economically unsustainable products. Similarly, without a viable business model, investors and pharmaceutical companies are unlikely to provide sufficient funding to bring the concept to market. Economic viability is therefore the first framework criterion, although it can be difficult to predict. For instance, in 2018, <2 years after gaining FDA approval, the animal pharmaceutical company that developed Imrestor®, an antibiotic alternative addressing mastitis in dairy cattle, decided to suspend its commercialization (7, 8).

To determine economic viability, animal health companies and investors evaluate the potential product's expected revenue and probability of success, compared to anticipated costs and risks (9). These evaluations usually take a global perspective, and factor in relevant national and regional policies, such as current or likely future antibiotic use restrictions and the broader regulatory landscape.

#### Expected Project Costs

The initial discovery and development of a new animal health product typically incurs substantial costs, as outlined in Table 1. Product manufacturing, service, distribution, disposal and extensions to new species or indications can constitute substantial additional costs which may be challenging to predict during initial development stages (9). Uncertainty in the predicted project cost and associated risks, including the probability of regulatory success or public acceptance, will also discourage investment.

#### Expected Product Revenue

To predict product revenue, investors analyze both the market and the product's expected performance in it. The predicted market size for an antibiotic alternative ultimately depends on the number of farms and animals affected by the disease and on how likely the producer or veterinarian is to proactively take steps to address it through prevention, control, or treatment. Economic factors play a role here as well, including short term disease impacts as well as long-term consequences on animal health and productivity (10, 11). Mastitis in dairy cattle, for instance, persistently decreases milk yields in subsequent lactations (12). Transboundary infectious animal diseases can also inflict additional economic costs, for instance through trade restrictions and loss of export markets. In contrast, some animal diseases are controlled most effectively through culling, and in certain cases the animal health benefits associated with an intervention may not outweigh the costs. Given the considerable R&D costs, to be economically viable, antibiotic alternatives for food producing species must address relatively common health problems (i.e., endemic infectious animal diseases) that have substantial economic and animal health impacts.

Other market characteristics, such as segmentation of the existing market and predicted market growth, factor into the revenue calculation as well. In addition, market access may be greater in more highly integrated industries and for products with more internationally harmonized regulatory requirements.

Product specific considerations include the expected return on investment (ROI) for the livestock producer, and the competitiveness of the product compared to alternatives. As outlined in Table 1, several factors impact the ROI, making it potentially challenging to predict (9, 13, 14). Product competitiveness refers to the availability of “substitutes”—interventions that address the same health issue. Particular attention is given to less expensive, easier to administer or more effective substitutes, which often include existing antibiotics (5). Expectation of equal or superior performance for alternatives compared to existing antibiotics may be unrealistic. However, increasing regulatory and market-based restrictions on antibiotic use may render even less-effective alternatives highly competitive. Increasing bacterial resistance to antibiotics among target pathogens may further reduce the efficacy of currently available antibiotics (15–17). Currently, few signs point to this phenomenon as an important driver of demand for antibiotic alternatives.

### Evaluating Project Risks

Even if these initial economic considerations are favorable, an antibiotic alternative candidate may fail for many reasons.

#### Product Safety

Product safety (see Table 1) is a prerequisite for the success of an antibiotic alternative and integral to the regulatory approval process but may be challenging to predict early in development. *In vitro* and *in silico* models have been developed to help assess the pharmacokinetics and predict the safety of veterinary drugs (18). The applicability of these models to antibiotic alternatives depends on the type of product, and can be influenced by the mechanism of action, host immune response, and potential for off-target effects. Ultimately, well-designed *in vivo* studies are critical for assuring end-users, regulators, and the public that a product is safe for animals, humans and the environment.

#### Product Efficacy

Antibiotic alternatives that do not meet customer expectations for efficacy in effect type—prevention, control and/or treatment,—as well as the magnitude and consistency of the effect, are unlikely to be successful. The mechanisms by which alternatives exert their effects are diverse: for instance, they may enhance host immunity, induce cytotoxic effects in pathogenic organisms, block proteins that mediate cell entry or virulence through passive immunization, promote gut health, exert anti-inflammatory properties, or modulate microbial communities in the gut (19–27). In general, the magnitude of the effect is lower for alternatives compared to antibiotics, and tends to be more variable across settings (5). Clarifying customer expectations around some minimum threshold for efficacy (for instance, compared to antibiotics) for the alternative product candidate may prove useful.

Predicting product efficacy early in R&D can be challenging. *In vitro* data are often used to predict efficacy because they are easier to collect and do not require the larger investments needed for *in vivo* studies (28–30). However, predictions based on these data are less reliable than *in vivo* studies, which better capture genetic differences between animals and variations in host-pathogen interactions and environment. Key design questions for studies of *in vivo* efficacy include whether diseases are experimentally introduced in healthy animals (i.e., challenge studies) or else the rates of natural disease occurrence are observed, and whether animals are managed under real world conditions (i.e., experimental vs. field trials). More tightly-controlled studies—such as those experimentally infecting a small number of healthy, genetically homogenous animals with one pathogen strain at one point in time, can use smaller experimental group sizes for statistical significance than less closely controlled studies, but they often do not adequately capture population-level variations that can impact efficacy. For instance, the experimental animals may be more uniform with regard to factors such as age, breed, health status, management, and disease history than animals in commercial settings (31). Study complexity and cost also limit the ability to evaluate efficacy under different animal housing and management practices.

For many antibiotic alternatives, conclusive data from large, well-controlled *in vivo* studies are scarce—an issue that is compounded by lack of information regarding the products' mechanism of action (22, 32–34). Potential interactions across alternatives and efficacy under varying management and husbandry practices have also remained largely unexplored (5). In the swine industry, for instance, a range of alternatives have been studied with mixed results, yet a systematic assessment of this body of research and a definitive conclusion of overall impact on swine health remains a major need (35, 36). When evaluating efficacy, it is important to recognize that many antibiotic alternatives stimulate host immunity broadly, or else alter the microbial environment to be less conducive to pathogen adhesion or propagation, rather than directly kill pathogens or inhibit their growth.

#### Product Acceptability

The acceptability of new alternatives by farmers and veterinarians, who often have vast experience using antibiotics, is also key to success. Studies have shown that many farmers and veterinarians are skeptical about the efficacy of antibiotic alternatives (37–39). Behavioral and socio-economic factors such as prior experience and risk avoidance clearly impact decision-making regarding the use of antibiotics or alternatives (40–42). Behavioral studies related to the use of antibiotics and other medications in human health care and animal agriculture have identified attitudes toward the product, belief in its value, and perceptions of behavioral constraints such as economics, risk, trust in others, social norms (i.e., expectations of others) and moral obligation to treat animals under one's care as core behavioral drivers (40–43). Building trust in a new product usually requires, at minimum, evidence of clear and consistent product efficacy under field conditions. Independent third-party verification, for instance as part of a data clearing-house or a trial registry, could help address concerns about data dredging and cherry-picking of efficacy trials, although it is unlikely to solve all the underlying challenges and concerns.

The success of an alternative also requires that the animal products derived using the alternative are acceptable to consumers. Generally speaking, the biological function of an alternative should be easy to explain to a layperson and must align with consumers' beliefs and expectations concerning food production and their conceptualizations of risk and adulteration. Alternatives may be preferable over antibiotics to some consumers, to the extent that they alleviate concerns regarding their use (44). In fact, some consumers perceive foods derived from animals raised without antibiotics as more healthful or nutritious (45). Ultimately, consumer acceptance of new technologies is often highly context-specific and affected by a variety of factors including moral, social, political, economic, and religious values as well as geographical, ecological, and animal welfare concerns (46).

#### Product Practicality and Ease of Use

The widespread adoption of an antibiotic alternative requires that they be practical to use for farmers and veterinarians. This means any such product must be integrated into current production practices without causing major disruptions. Products that require disruptive shifts in the infrastructure or systems under which livestock commodities are raised are unlikely to succeed, at least in the shorter- to medium-term. In addition, products that are not readily compatible with current animal production practices—for instance, because of their application frequency, mode of administration, stability or timing of use—may face obstacles to adoption. Side-effects of the product or the stress associated with handling an animal to apply the product may also reduce adoption. As with antibiotics, farmers may need to observe specific withdrawal times that may limit their ability to market animal-derived products.

## Discussion

Antibiotic alternatives represent a major unmet need for the livestock sector. However, the factors predicting their success or failure are complex. Here, we outline a framework for the evaluation of alternative candidates that may empower federal agencies, philanthropic organizations, and other key stakeholders to consistently and transparently prioritize investments in antibiotic alternatives. Our framework first considers the overall costs and benefits related to the new alternative, because economic viability is foundational to ultimate commercial success and this information may be readily available prior to or early during R&D.

Ultimately, bringing an alternative to market is an extremely complex process, involving evaluation of product safety, efficacy, acceptability and practicality. Therefore, the potential success of a new alternative may be best evaluated from multiple perspectives, an approach that we replicated in our original survey and workshop design and encourage in the evaluation of alternatives. Research funders may, for instance, start to involve farmers, veterinarians and farm advisors more closely in early funding decisions. Developing new antibiotic alternatives is a challenging issue but holds considerable promise for animal health and the fight to combat antibiotic resistance. This framework will empower research funders to evaluate alternatives early during R&D, and to dedicate scarce funding to the most promising alternatives.

## Data Availability Statement

The raw data supporting the conclusions of this manuscript will be made available by the authors, without undue reservation, to any qualified researcher.

## Author Contributions

All authors listed have made a substantial, direct and intellectual contribution to the work, and approved it for publication.

### Conflict of Interest

The authors declare that the research was conducted in the absence of any commercial or financial relationships that could be construed as a potential conflict of interest.

## Footnotes

^1^Note that we use the term antibiotic in this paper to be consistent with common industry language and to avoid confusion with those alternatives that are antimicrobials; however, it is important to note that the terms often are used interchangeably, though technically antibiotics are a subset of antimicrobials.

^2^https://www.cdc.gov/antibiotic-use/stewardship-report/hospital.html

^3^https://www.ars.usda.gov/alternativestoantibiotics/Symposium2016/ATAWorkshop2016.html
